# Medical Schools for Women

**Published:** 1896-09-05

**Authors:** 


### Medical Schools for Women.

The following medical schools are open for women, in connection with each of which arrangements are made for their students obtaining hospital practice and clinical instruction:—

London.—The London School of Medicine for Women, the composition fee of which is £125; or first year, £40; second year, £40; third year, £30; fourth year, £25.

Newcastle-on-Tyne. — The Medical and Science Faculties of the University of Durham. For fees see page 374

Edinburgh.—The Edinburgh School of Medicine for Women, and the Medical College for Women, Edinburgh. Total fees for school and hospital, £100 in one sum; or £105 in instalments.

Glasgow.—Queen Margaret College, in connection with the Glasgow University.

St. Andrew's.—Certain courses are open to women the rest being given by lecturers of the Edinburgh School of Medicine.

Dundee.—All the courses which are given, as well as the hospital practice, are open to women.

Dublin.—The Schools of Surgery of the Royal College of Surgeons of Ireland are open to women students, for whose use special rooms are set apart. A complete medical education is given.

